# Does socioeconomic and environmental burden affect vulnerability to extreme air pollution and heat? A case-crossover study of mortality in California

**DOI:** 10.1038/s41370-024-00676-9

**Published:** 2024-05-07

**Authors:** Mehjar Azzouz, Zainab Hasan, Md Mostafijur Rahman, W. James Gauderman, Melissa Lorenzo, Frederick W. Lurmann, Sandrah P. Eckel, Lawrence Palinkas, Jill Johnston, Michael Hurlburt, Sam J. Silva, Hannah Schlaerth, Joseph Ko, George Ban-Weiss, Rob McConnell, Leo Stockfelt, Erika Garcia

**Affiliations:** 1 Occupational and Environmental Medicine, School of Public Health and Community Medicine, Institute of Medicine, Sahlgrenska Academy, University of Gothenburg, Gothenburg, Sweden; 2 Department of Occupational and Environmental Medicine, Sahlgrenska University Hospital, Gothenburg, Sweden; 3 Department of Population and Public Health Sciences, Keck School of Medicine, University of Southern California, Los Angeles, CA USA; 4 Department of Environmental Health Sciences, Tulane University School of Public Health and Tropical Medicine, New Orleans, LA USA; 5 Sonoma Technology, Inc., Petaluma, CA USA; 6 Herbert Wertheim School of Public Health and Human Longevity Science, University of California San Diego, La Jolla, CA USA; 7 Suzanne Dworak Peck School of Social Work, University of Southern California, Los Angeles, CA USA; 8 Department of Civil and Environmental Engineering, Viterbi School of Engineering, University of Southern California, Los Angeles, CA USA; 9 Department of Earth Sciences, University of Southern California, Los Angeles, CA USA; 10 Columbia Climate School, Columbia University, New York, NY USA

**Keywords:** Air pollution, Particulate matter, Vulnerable populations, Climate change, Environmental justice, Epidemiology

## Abstract

**Background:**

Extreme heat and air pollution is associated with increased mortality. Recent evidence suggests the combined effects of both is greater than the effects of each individual exposure. Low neighborhood socioeconomic status (“socioeconomic burden”) has also been associated with increased exposure and vulnerability to both heat and air pollution. We investigated if neighborhood socioeconomic burden or the combination of socioeconomic and environmental exposures (“socioenvironmental burden”) modified the effect of combined exposure to extreme heat and particulate air pollution on mortality in California.

**Methods:**

We used a time-stratified case-crossover design to assess the impact of daily exposure to extreme particulate matter <2.5 μm (PM_2.5_) and heat on cardiovascular, respiratory, and all-cause mortality in California 2014–2019. Daily average PM_2.5_ and maximum temperatures based on decedent’s residential census tract were dichotomized as extreme or not. Census tract-level socioenvironmental and socioeconomic burden was assessed with the CalEnviroScreen (CES) score and a social deprivation index (SDI), and individual educational attainment was derived from death certificates. Conditional logistic regression was used to estimate associations of heat and PM_2.5_ with mortality with a product term used to evaluate effect measure modification.

**Results:**

During the study period 1,514,292 all-cause deaths could be assigned residential exposures. Extreme heat and air pollution alone and combined were associated with increased mortality, matching prior reports. Decedents in census tracts with higher socioenvironmental and socioeconomic burden experienced more days with extreme PM_2.5_ exposure. However, we found no consistent effect measure modification by CES or SDI on combined or separate extreme heat and PM_2.5_ exposure on odds of total, cardiovascular or respiratory mortality. No effect measure modification was observed for individual education attainment.

**Conclusion:**

We did not find evidence that neighborhood socioenvironmental- or socioeconomic burden significantly influenced the individual or combined impact of extreme exposures to heat and PM_2.5_ on mortality in California.

**Impact:**

- We investigated the effect measure modification by socioeconomic and socioenvironmental of the co-occurrence of heat and PM_2.5_, which adds support to the limited previous literature on effect measure modification by socioeconomic and socioenvironmental burden of heat alone and PM_2.5_ alone. We found no consistent effect measure modification by neighborhood socioenvironmental and socioeconomic burden or individual level SES of the mortality association with extreme heat and PM_2.5_ co-exposure. However, we did find increased number of days with extreme PM_2.5_ exposure in neighborhoods with high socioenvironmental and socioeconomic burden. We evaluated multiple area-level and an individual-level SES and socioenvironmental burden metrics, each estimating socioenvironmental factors differently, making our conclusion more robust.

## Background

Climate change has been recognized as the world’s greatest public health threat [1]. There is ample evidence that climate change will increase the intensity and frequency of heat waves around the world [2–5]. Many studies have shown associations between exposure to high temperatures and heat waves and increased mortality in cardiovascular (CV) and respiratory diseases [6–8]. Extreme heat can directly contribute to death (e.g., through heat stress); at the same time, deaths attributable to heat exposure are related to worsening of pre-existing medical conditions such as CV disease, pulmonary disease, and kidney disorders [9]. Increased ambient particulate matter (PM) is another important factor being impacted by climate change [3]. Health effects of fine particulate matter (PM_2.5_) are well documented, including deterioration of lung function, exacerbation of respiratory conditions such as asthma [10], increased risk of CV diseases [11], and cardiorespiratory mortality [12, 13]. Wildfires are becoming more frequent in many areas, and can be an important source of increased PM < 2.5 μm (PM_2.5_) pollution in both urban and rural communities [14]. Wildfire-related PM_2.5_ pollution has significant effects on respiratory [15] and CV health [16]. While there are several studies discussing the separate effects of heat and air pollution on mortality [17, 18], few studies have looked at the interaction effects of heat and air pollution [19, 20]. We recently evaluated the mortality effects of extremes of heat and air pollution in California and found that co-occurrence of extreme heat and PM_2.5_ had higher mortality risk than exposure to either extreme heat or PM_2.5_ alone [21].

The relationships between heat and air pollution exposure, socioeconomic disparities, and adverse health effects are complex. There is evidence showing low socioeconomic status (SES) communities have a higher health burden due to ambient PM pollution and heat both from increased exposure and increased vulnerability to exposure [22–26]. Furthermore, these effects may be influenced by other environmental co-exposures. For example, living close to major roads will lead to exposure to both air pollution and noise from traffic, and a lack of green space in the residential area (which could be seen as an environmental risk factor by itself) can be associated with both higher air pollution exposure and an increased vulnerability to heat [25, 27, 28]. The relationships are contextual, however, and may differ between different areas. There is thus a need to investigate how higher “socioenvironmental” burden influences the vulnerability to environmental exposures [25].

Communities with low SES are sometimes referred to as environmental justice (EJ) communities, because they are burdened with both increased environmental exposures and decreased protection connected to their lower SES [29]. Understanding the effect that historical social and economic marginalization has on the mortality risks from extreme heat and air pollution is critical given the disproportionate environmental health burdens and vulnerabilities experienced by EJ communities and the projected increases in these exposures with progressing climate change [30, 31]. The objective of this study was to evaluate whether neighborhood socioeconomic or socioenvironmental status (proxies for barriers resulting from historical socioeconomic marginalization) modifies the mortality association with co-exposure to extreme heat and PM_2.5_, extending our prior work on these exposures [21]. We investigated the impact of exposure to extreme heat and PM_2.5_ alone and in combination. We investigated effect measure modification for all-cause, CV, and respiratory mortality, based on neighborhood socioenvironmental and socioeconomic burden as well as individual vulnerability.

## Methods

### Study population

Mortality data for California from January 1, 2014 to December 31, 2019 were obtained from the California Department of Public Health’s Vital Statistics. We obtained data on a total of 1,580,799 deaths that occurred in California during the study period. We excluded 51,557 deaths (3.4%) that were missing residential census tract and an additional 14,950 deaths (0.9%) with a residential census tract outside of California, because California-based census tract was needed for assessment of exposures and burden metrics. We had a final sample of 1,514,292 deaths. All-cause mortality included all deaths and cause-specific mortality were defined using reported international classifications of disease, tenth edition (ICD-10) codes for cause of death, including CV (I10-I70) and non-malignant respiratory (J00-J99) mortality. Demographic characteristics of decedents were obtained from death certificates, including residential census tract, highest educational attainment, age, sex, race, and ethnicity.

### Exposure assessment

Rahman et al. described the assessment and parameterization of exposure to extreme PM_2.5_ and extreme temperature. Briefly, monitored PM_2.5_ concentration data were obtained from the US Environmental Protection Agency’s Air Quality System [32]. Daily (24-h) PM_2.5_ concentrations were estimated for all California census tracts (population-weighted centroids) using inverse distance-squared weighting of observations from up to four nearby monitoring locations within a 50 km radius [33]. Maximum daily temperature and relative humidity were assessed using nearest grid based on a 4-km gridded reanalysis dataset estimating daily meteorological conditions, developed at the University of Idaho [34]. PM_2.5_ and temperature exposures were assigned based on decedent’s residential census tract for each of the same day (lag0) and one day before (lag1).

Extreme exposure was defined as exposure above the 90th, 95th, or 97th percentile for either heat or PM_2.5_. The percentile distribution for heat was specific to each census tract and included all daily temperatures across the study period (2014–2019) for a given census tract. The percentile distribution for PM_2.5_ concentrations included all daily mean PM_2.5_ across the study period for all census tracts in California—because unlike temperature there is little evidence that people can acclimate to local levels of PM_2.5_. Exposure was parameterized as either a day with (i) extreme PM_2.5_ only, (ii) extreme heat only, (iii) both extreme PM_2.5_ and heat, or (iv) neither extreme PM_2.5_ nor heat.

### Socioenvironmental and socioeconomic burden data

Data on census tract socioenvironmental burden was obtained from CalEnviroScreen, an open access tool developed by the California Office of Environmental Health Hazard Assessment to identify communities that are exposed to many sources of environmental burdens and also communities which are the most vulnerable to these exposures [35]. The CalEnviroScreen score (CES) is a measure of each census tract’s socioeconomic and cumulative environmental burden and is composed of two components: the socioeconomic score and the environmental score. The socioeconomic score includes indicators such as unemployment, education, poverty and long-term health burden while the environmental score includes for example exposure to hazardous waste, drinking water contamination, toxic release from facilities, and long-term air pollution. Indicators used for the two components are presented in Supplementary Table S1. The percentiles for the individual indicators were averaged, creating the score for the component. The components were then used to calculate the CES as a value from 0–100, where a higher score indicates a greater socioenvironmental burden. Previous literature has used the CES to study the effect of higher socioenvironmental burden on health [23, 36, 37]. Most data for indicators were collected between 2015–2021 (Supplementary Table S1).

We used a census tract Social Deprivation Index (SDI) developed by the Robert Graham Center that combines seven indicators from the 2011–2015 American Community Survey 5-year estimates data to reflect census tract level social inequalities [38]. The index includes indicators such as education, income, housing characteristics and percentage in the population that are high needs. Descriptions of the seven indicators are presented in Supplementary Table S2. SDI ranged from 1 to 100 with a higher SDI indicating a higher level of social deprivation.

The Healthy Places Index (HPI), developed by the Public Health Alliance of Southern California, is an open access tool for exploring local factors and determinants of health at the census tract level [39], and was used as an alternative measure for socioenvironmental burden in a sensitivity analysis. The HPI considers several indicators, including education, housing, voting registration, long-term environmental exposure, and transportation. The HPI uses data from the 2015–2019 American Community Survey 5-year estimates, US EPA, and Comprehensive Housing Assessment System 2014–2018 [39].

All of the above-mentioned area-level measures of socioenvironmental or socioeconomic burden were available at the census tract level and were linked to decedents based on their residential census tract. We additionally evaluated an individual-level marker for socioeconomic status: educational attainment as reported on death certificates. Educational attainment categorized individuals above the age of 25 as those with a high school diploma and those without a high school diploma, mirroring the index CalEnviroScreen uses for educational attainment.

### Data analysis

A time-stratified case-crossover study design was used to assess associations between exposure to extremes of PM_2.5_ and heat and odds of all-cause, CV, and respiratory mortality in California during the years 2014–2019. For each decedent, the date of death served as the case day, to which we matched control days defined as days occurring within the same month and year and falling on the same day of the week as the case day [40]. This design inherently controls for potential confounding effects of individual-level time-independent characteristics (e.g., sex, ethnicity, socioeconomic status) as well as day of the week, long-term trend, and seasonality [41].

We used conditional logistic regression models adjusted for relative humidity, included as a natural cubic spline with three degrees of freedom. In our previous paper [21] we investigated the association of extreme heat and air pollution alone and combined extreme heat and air pollution exposure with all-cause, cardiovascular and respiratory mortality. This paper introduced product terms into all our models to study effect measure modification of the extreme exposure and mortality relation by neighborhood socioenvironmental (i.e., CES) or socioeconomic (i.e., SDI) burden, categorized into quartiles or a binary variable based on the sample (or subsample for cause-specific mortality) distribution.

For all-cause mortality we performed analyses using the 90th, 95th, and 97th percentile thresholds as extreme exposure, and a product term between the extreme exposures and quartiles of the CES and SDI. For cardiovascular mortality we performed analyses using the 90th percentile threshold as extreme exposure, and a product term between the extreme exposure and quartiles of the CES and SDI. For respiratory mortality we performed analyses using the 90th percentile threshold as extreme exposure, and a product term between the extreme exposure and a binary variable (above/ below median) for the CES and SDI.

All extreme exposures used the same thresholds, i.e. for analyses with extreme exposure above the 90th percentile, extreme heat and air pollution alone and combined extreme heat and air pollution exposure all used the 90th percentile threshold. For CV and respiratory mortality, only the 90th percentile threshold for extreme exposure was assessed due to smaller sample sizes in the other percentile thresholds. The limited sample size for respiratory mortality was also the reason the CES and SDI were introduced into the models as product terms with binary variables instead of quartiles. In the crossover study design, no main effect of the neighborhood characteristic on mortality could be estimated. Our main models for all mortality outcomes evaluated same day (lag-0) PM_2.5_ and heat exposure. In our previous paper mortality effects of combined heat and PM_2.5_ exposure were primarily observed for lag-0 (now used in our main analyses) and lag-1 (now used in a sensitivity analysis) [21]. Secondary analyses were conducted: (i) evaluating individual level educational attainment as an effect measure modifier (excludes deaths with missing educational attainment), and (ii) using prior day (lag-1) exposure with the 90th percentile threshold.

We performed several sensitivity analyses assessing effect measure modification by alternative neighborhood socioeconomic/environmental burden metrics, all using same lag-0 exposure and extremes defined using the 90th percentile threshold: 1) the socioeconomic burden score component of the CES; 2) the environmental burden score component of the CES; and 3) the HPI. We also performed a sensitivity analysis using continuous temperature and PM_2.5_ exposure from the summer months to investigate possible effect measure modification with non-extreme exposure. These were modeled similarly to the models described above, but in lieu of days being categorized based on extreme exposures, linear terms for temperature, PM_2.5_, and a linear interaction term between the two exposures were included to capture temperature and PM_2.5_ exposures individually as well as their co-exposure. Effect measure modification by CES or SDI was then evaluated using product terms between these continuous exposures and quartile of CES/SDI. All analyses were performed in R [42]. Participants with missing PM_2.5_, heat, or humidity data possibly due to missing residential information were excluded from the analysis.

## Results

Study population demographics are presented in Table 1. The study included 1,514,292 cases of all-cause deaths, of which 33% were CV and 9% were respiratory. Differences in sociodemographic variables were similar for those with high CES and high SDI, compared to low CES/SDI. The mean age at death was 74.0 years, with a lower age at death for decedents living in census tracts with higher CES and SDI. Furthermore, those with higher CES and SDI consisted proportionally more of Black and Hispanic populations and fewer White populations. Lower CES and SDI were observed among decedents with higher educational attainment. The average daily maximum temperature for those exposed to extreme (>90th percentile) heat alone and to combined extreme heat and extreme PM_2.5_ was 34.6 and 36.7° Celsius, respectively, while the average daily PM_2.5_ exposure for those exposed to extreme PM_2.5_ alone and to combined extreme heat and PM_2.5_ was 26.7 and 24.2 μg/m^3^, respectively. Exposure to extreme PM_2.5_ levels increased with higher CES and SDI (6 and 8% of days had extreme PM_2.5_ levels in the low CES and SDI group respectively and 12 and 11% of days had extreme PM_2.5_ levels in the high CES and SDI group respectively). Similar patterns were observed when extreme exposure was defined using 95th and 97th percentile thresholds (Supplementary Table S3).

**Table 1:** Demographic characteristics of the study population overall and by socio-environmental and socioeconomic burden.

	All	Socio-environmental burden		Socioeconomic burden		Missing (*n*)
		Low^a^	High	Low^a^	High	
Number of cases, *n* (%)						0
All-cause	1,514,292 (100%)	758,217 (100%)	756,036 (100%)	760,424 (100%)	753,715 (100%)	
Cardiovascular	492,513 (33%)	249,723 (33%)	242,772 (33%)	246,294 (32%)	246,157 (33%)	
Respiratory	139,116 (9%)	70,088 (9%)	69,025 (9%)	69,045 (9%)	70,061 (9%)	
Age at death, years	74.0 (18)	76.7 (17)	71.4 (19)	76.8 (17)	71.2 (19)	86
Sex, *n* (%)						30
Male	780,835 (52%)	381,336 (50%)	399,499 (53%)	384,177 (51%)	396,582 (53%)	
Female	733,426 (48%)	374,692 (50%)	358,734 (47%)	376,242 (49%)	357,107 (47%)	
Race/Ethnicity, *n* (%)						16,060
White^b^	928,651 (61%)	574,446 (76%)	354,205 (47%)	563,922 (74%)	364,610 (48%)	
Black	114,044 (8%)	26,620 (4%)	87,424 (12%)	29,209 (4%)	84,825 (11%)	
American Indian/Alaskan^b^ Native	8049 (0.5%)	3615 (0%)	4434 (1%)	2911 (0.4%)	5137 (0.7%)	
Asian/Hawaiian/Pacific Islander	154,882 (10%)	72,043 (10%)	82,839 (11%)	76,428 (10%)	78,444 (10%)	
Hispanic	289,246 (19%)	69,946 (9%)	219,300 (29%)	79,115 (10%)	210,119 (28%)	
Multiracial/Other	19,420 (1%)	9366 (1%)	10,054 (1%)	8839 (1%)	10,580 (1%)	
Education, *n* (%)						36 460
Less than High School	303,230 (20%)	89,226 (12%)	214,004 (28%)	91,405 (12%)	211,811 (28%)	
High School	520,270 (34%)	252,917 (33%)	267,353 (35%)	253,597 (33%)	266,624 (35%)	
Some college	249,177 (16%)	136,248 (18%)	112,929 (15%)	136,531 (18%)	112,614 (15%)	
University degree	405,155 (27%)	266,055 (35%)	139,100 (18%)	268,163 (37%)	136,938 (22%)	
90^th^ percentile threshold extreme exposure^c^						
*Extreme heat only*						
Case days, n (%)	133,196 (9%)	67,480 (9%)	65,716 (9%)	67,161 (9%)	66,027 (9%)	0
Temperature^d^, Celsius	34.6 (4)	34 (4)	35.3 (4)	34.1 (4)	35.1 (4)	0
*Extreme PM*_*2.5*_ *only*						
Case days, *n* (%)	140,197 (9%)	48,944 (6%)	91 253 (12%)	58,684 (8%)	81,493 (11%)	0
PM_2.5_, μg/m^3^	26.7 (16.2)	26.7 (19)	26.8 (15)	26.2 (17)	27.1 (16)	0
*Extreme heat and PM*_*2.5*_						
Case days, *n* (%)	11,306 (1%)	4493 (1%)	6813 (1%)	5152 (1%)	6153 (1%)	0
Temperature^d^, Celsius	36.7 (4)	36.1 (4)	37.1 (3.5)	36.4 (4)	37.0 (4)	0
PM_2.5_ levels, extreme^c^ heat and PM_2.5_, μg/m^3^ mean (SD)	24.2 (10.1)	25.6 (12.2)	23.3 (8.3)	24,4 (11)	23.9 (10)	0
Relative humidity, %	80.4 (13.5)	81.3 (13.8)	79.5 (13)	80.3 (18)	80.7 (17)	0
SDI/ Socioeconomic burden	58 (28)	38.9 (20)	76.5 (17)	33.4 (17)	82.2 (12)	153
CalEnviroScreen score)						1 561
Socioenvironmental burden	27.4 (15.7)	14.6 (3.1)	40.2 (6.4)	17.3 (10)	37.6 (14)	1 561
Socioeconomic score	5.1 (1.5)	3.6 (1.2)	6.7 (1.1)	4.6 (1)	5.6 (1)	1 561
Environmental score	5.1 (2.1)	4.2 (1.1)	6.1 (1)	3.7 (1)	6.6 (2)	0
HPI score	0.0014 (0.52)	0.4 (0.4)	−0.3 (0.4)	0.38 (0.3)	−0.38 (0.4)	10 652

Mean (standard deviation), unless otherwise noted.

^a^Categorization was defined based on the distribution of the burdens in the population. Below/ above a CalEnviroScreen score of 24.7 (median) for low/high socioenvironmental burden and below/ above a Social Deprivation of 60 (median) for low/ high socioeconomic burden.

^b^Non-Hispanic.

^c^Extreme exposures thresholds were defined based on distributions across the entire study period within census tract [temperature] or across the state [PM_2.5_].

^d^Average maximum daily temperature.

**Fig. 1:**
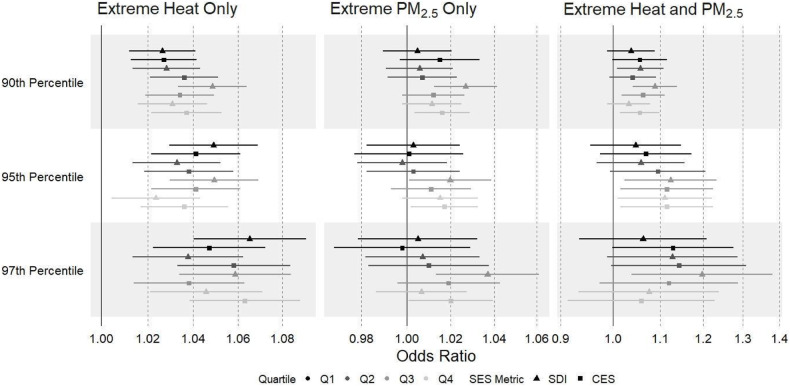
All-cause mortality and extreme heat and PM_2.5_ by socioenvironmental (CES) or socioeconomic (SDI) burden. Association between all-cause mortality and extreme heat alone, extreme PM_2.5_ alone, and combined extreme heat and PM_2.5_ by quartiles of socioenvironmental (CES) or socioeconomic (SDI) burden (with 95% confidence intervals). Models were fitted separately for CES and SDI using conditional logistic regression adjusted for relative humidity as natural cubic spline with main effects for the extreme exposure category (neither [referent], heat only, PM_2.5_ only, or both heat and PM_2.5_) and a product term between extreme exposure category and quartile of CES or SDI to obtain burden-quartile-specific Odds Ratios. Extreme exposure was defined as over the 90th, 95th and 97th percentile in separate models. Note: axes scales are different between exposures.

The product terms and CES and SDI stratum-specific confidence intervals for the odds ratios associated with extreme exposures >90th percentile, >95th percentile and >97th percentile on all-cause mortality are presented in Fig. 1 and in detail in Supplementary Table S4. As previously reported, there were statistically significant associations between extreme heat and air pollution alone and combined extreme heat and air pollution exposure and all-cause, cardiovascular and respiratory mortality [21]. Although extreme heat and air pollution exposure alone was statistically significantly associated with increased mortality, exposure to combined extreme heat and air pollution had consistently stronger mortality odds ratios compared to extreme heat and air pollution alone.

**Fig. 2:**
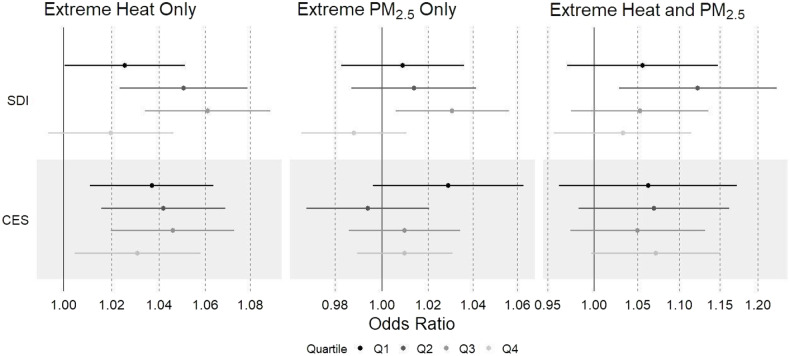
Cardiovascular mortality and extreme heat and PM_2.5_ by socioenvironmental (CES) or socioeconomic (SDI) burden. Association between CV mortality and extreme heat alone, extreme PM_2.5_ alone, and combined extreme heat and PM_2.5_ by quartiles of socioenvironmental (CES) or socioeconomic (SDI) burden (with 95% confidence intervals). Models were fitted separately for CES and SDI using conditional logistic regression adjusted for relative humidity as natural cubic spline with main effects for the extreme exposure category (neither [referent], heat only, PM_2.5_ only, or both heat and PM_2.5_) and a product term between extreme exposure category and quartile of CES or SDI to obtain burden-quartile-specific Odds Ratios. Extreme exposure was defined as over the 90th percentile. Note: axes scales are different between exposures.

**Fig. 3:**
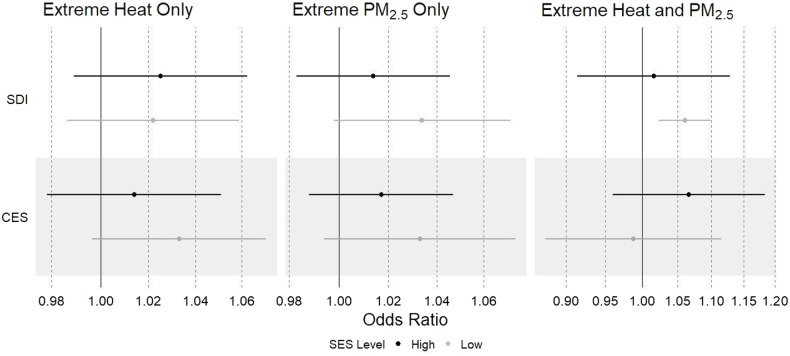
Respiratory mortality and extreme heat and PM_2.5_ by socioenvironmental (CES) or socioeconomic (SDI) burden. Associations between respiratory mortality and extreme heat alone, extreme PM_2.5_ alone, and combined extreme heat and PM_2.5_ by binaries of socioenvironmental (CES) or socioeconomic (SDI) burden (with 95% confidence intervals). Models were fitted separately for CES and SDI using conditional logistic regression adjusted for relative humidity as natural cubic spline with main effects for the extreme exposure category (neither [referent], heat only, PM_2.5_ only, or both heat and PM_2.5_) and a product term between extreme exposure category and binaries of CES or SDI to obtain burden-binary-specific Odds Ratios. Extreme exposure was defined as over the 90th percentile. Note: axes scales are different between exposures.

However, there was no consistent pattern of either increasing or decreasing mortality effect (all-cause, CV or respiratory) estimates of combined heat and PM_2.5_ effect across census tract CES or SDI. Similar null modifying effects of CES and SDI were observed for extreme heat and PM_2.5_ exposure separately (Fig. 1 and Supplementary Table S4), and for cause-specific CV and respiratory mortality (Figs. 2 and 3, respectively, and Supplementary Table S4). There was statistically significant positive effect measure modification in the third quartile of SDI on the all-cause and CV mortality odds ratio of extreme heat alone, as well as on the all-cause mortality odds ratio of extreme PM_2.5_ alone (Supplementary Table S4). However, this was not observed in other quartiles or when 95th or 97th percentiles of extreme exposure were used.

**Fig. 4:**
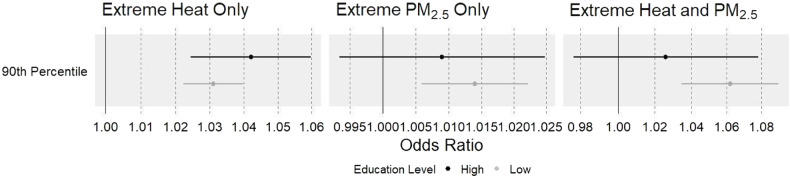
All-cause mortality and extreme heat and PM_2.5_ by individual socioeconomic (SDI) burden. Associations between all-cause mortality and extreme heat alone, extreme PM_2.5_ alone, and combined extreme heat and PM_2.5_ by high and low education (with 95% confidence intervals). Models were fitted with educational attainment using conditional logistic regression adjusted for relative humidity as natural cubic spline with main effects for the extreme exposure category (neither [referent], heat only, PM_2.5_ only, or both heat and PM_2.5_) and a product term between extreme exposure category and educational attainment to obtain educational attainment-specific Odds Ratios. Extreme exposure was defined as over the 90th percentile. Note: axes scales are different between exposures.

Secondary and sensitivity analyses also did not find strong evidence supporting effect measure modification by SES. There was no statistically significant effect measure modification by individual-level educational attainment (Fig. 4 and Supplementary Table S4). Analyses using lag-1 exposure instead of lag-0, the socioeconomic and environmental burden components of the CES, the HPI, or using continuous instead of categorical extreme heat and PM_2.5_ exposure had similar null results with no consistent evidence for effect measure modification by these factors (Supplementary Table S4 and Supplementary Figs. S1 and S2). There was some indication of effect measure modification by SDI on the mortality odds ratio associated with a continuous measure of PM_2.5_. The PM_2.5_ mortality odds ratio in the second and third quartile of SDI was slightly increased compared with the PM_2.5_ mortality effect in the first quartile of SDI, but no statistically significant difference in the fourth quartile, and no evidence of effect measure modification for either continuous heat or the heat-PM_2.5_ interaction effect.

## Discussion

In this time-stratified case-crossover study we found no consistent effect measure modification by neighborhood socioenvironmental and socioeconomic burden on the mortality effect of exposure to extremes of heat alone, PM_2.5_ alone, or co-occurrence of extreme heat and PM_2.5_ in California. There was no consistent pattern in the estimated associations with more socioeconomic burden. Results were similarly null for the effect of socioenvironmental and socioeconomic burden on the association of heat and PM_2.5_ with CV and respiratory mortality. Null results also were observed when individual-level educational attainment as socioeconomic status was used instead of census tract level measures as effect measure modifiers. Sensitivity analyses using lag-1 exposure instead of lag-0, alternative metrics for neighborhood socioeconomic or socioenvironmental burden, and continuous exposure analysis also produced null results. Similar to our previous study in this data set, there were statistically significant associations between extreme heat and air pollution alone and combined extreme heat and air pollution exposure and mortality [21].

Previous research has investigated how socioeconomic factors may modify the health effects of heat and air pollution exposure, with inconsistent results [42–45]. To our knowledge, no prior study has analyzed the modifying effect of socioeconomic or socioenvironmental factors on the combined impacts of heat and air pollution, making it challenging to directly compare our results to previous studies. While effect measure modification has not been evaluated for the combined impact of heat and PM_2.5_, there are studies on these exposures separately. However, it is important to keep in mind that in the present study combined extreme heat and PM_2.5_ was treated as its own exposure category separate from days with extreme heat alone and extreme air pollution alone. Thus, results may not be directly comparable to studies not separating these exposures.

A systematic review by Laurent et al. [43] on whether SES was an effect measure modifier of air pollution mortality effects identified 15 studies investigating short-term air pollution exposure and SES. A main conclusion of this work was that the resolution of the socioeconomic variable was an important factor. Studies using individual level data observed some statistically significant, albeit not uniformly consistent, results. Studies with SES data only at the area-level resolution (e.g., county or city) mostly found no statistically significant modifying effects. Only the study by Martins et al. [46] which studied modifying effects of area-level (city-subdivision) socioeconomic variables on the association between particulate matter <10 μm (PM_10_) exposure and respiratory mortality,found that people in areas with lower educational level and income had larger PM <10 μm effect estimates. We found no statistically significant results with area-level metrics of SES, for either combined co-occurring heat and PM_2.5_ or for heat and PM_2.5_ separately, or with an individual level measure of SES. It is possible that our use of categorized educational attainment (with or without a high school diploma) was too crude a measure of individual SES.

More recent studies since the systematic review using area-level measures have found statistically significant effect measure modification of PM effects. A cross-sectional study by Bevan et al. [47] examining the association between PM_2.5_ and CV mortality at the county-level by SDI in the US found a statistically significant positive effect measure modification between PM_2.5_ and SDI, indicating that areas with higher social deprivation were more vulnerable to the CV mortality effects of PM_2.5_. Another study by Jones et al. [16] investigated the association between wildfire-related PM and out-of-hospital cardiac arrests in California. An analysis stratifying the study population by high/low neighborhood-SES (categorized as low SES if more than 20% of the census tract population lived in poverty) found that in general the low SES population had higher effect estimates. Although our study did not directly study wildfire related PM, days with extreme heat and PM_2.5_ exposure in our study had substantial overlap with wildfire days [21]. These studies’ findings contrast with our findings of no effect measure modification by neighborhood SES on PM_2.5_-mortality associations.

Previous research investigating effect measure modification by SES of heat-related mortality also found inconsistent results. A study by Chan et al. [45] on the intra-city variability of the heat-mortality association in Hong Kong reported larger effect estimates in neighborhoods with lower income, although they only conducted stratified analysis and effect estimates were non-significant in all strata (low, medium, high). A case-crossover study by Xu et al. [44] investigating differences in heat-mortality effects found that Brazilian cities with lower literacy rates, income, and urbanization had statistically significant higher effect estimates. Our study in California, on the contrary, did not observe any consistent statistically significant effect measure modification by SES or socioenvironmental scores for heat-mortality associations.

Higher levels of daily PM_2.5_ exposure were observed with increasing CES and SDI, however. This aligns with previous reports in the U.S., including California, noting that air pollution levels are higher in areas with historically marginalized populations [48–50]. There are multiple mechanisms through which socioeconomic status might modify the health effects of heat and air pollution, but there are two that are particularly relevant to our study. First, exposure levels might be higher, as observed in the current study (Table 1), which could lead to larger health effects. Second, the vulnerability to exposure might be higher due to preexisting conditions, lifestyle factors, and increased exposure to other environmental sources, i.e., cumulative exposure [23, 24, 48]. For example, the prevalence of CV disease and diabetes are considerably higher among people with lower SES, and these conditions increase vulnerability to PM_2.5_ and heat [51, 52]. Differences in smoking habits across SES groups may increase vulnerability to air pollution effects [53]. Our study was focused on the second mechanism, but did not observe any increased vulnerability.

We used multiple area-level socioeconomic and environmental burdens metrics to gain a fuller understanding of effect measure modification by these burdens. The different metrics all produced null results. These indices were created to enable comparison between census tracts and were not primarily designed to be used as potential effect measure modifiers in studies with short-term exposures. To gain a broader understanding of effect measure modification by socioenvironmental and socioeconomic burden, initiatives to produce up-to-date data on environmental and socioeconomic factors may be beneficial, as most measures used 5-year averages or data from the last available year, which could vary significantly between different socioeconomic and environmental factors. These types of data might not be well-suited for studies like ours [35, 39, 54].

This study has some limitations. First, we used residential census tract and inverse distance-squared weighting of nearby air monitoring locations to assign exposure as opposed to residential or personal heat and PM_2.5_ exposure, which might introduce exposure measurement error. We did not have access to another source of air pollution data, such as modeled gridded predictions of air pollution exposure. However, leave-one-out evaluation of the inverse distance-squared method in California indicated small biases (<0.7 ppb and <0.5 μg/m^3^) and acceptable mean errors (<35%) [55]. Additionally, although people usually spend most of their time at home, we did not take into account exposure participants may have experienced while in transport, working, and elsewhere [56]. Secondly, we were not able to account for air conditioning (AC) use. The use of AC is closely connected to vulnerability as it decreases indoor exposure to both heat and air pollution and is related to socioeconomic status, especially income [22]. AC use data have been a limitation of many studies on health effects related to heat/ heat waves, especially since effects may vary substantially depending on AC use [22]. Nonetheless, our prior study using the same strategy observed associations between heat and PM_2.5_ and mortality using this exposure assessment method Rahman et al. [21], suggesting these were likely not a major source of bias. Third, decedents needed to have residential information to be assigned exposure. Those without residential data were excluded. People experiencing homelessness likely were in this group and they tend to experience a high burden of socioenvironmental factors [57, 58], thus we might be excluding this potentially highly vulnerable group. Fourth, we only had access to educational attainment from death certificates as a measure of individual-level SES, which might have been too crude of a proxy for this complex factor. Fifth, a consequence of only using extreme exposure is that the proportion of exposed to extreme exposure will have a maximum prevalence of 10%, when using extremes over the 90th percentile. In general, analyzing effect measure modification requires more power compared to finding a main effect [59]. Future studies could increase power by using a longer time period or all census tracts in the US. Sixth, California is a diverse region with a wide range of climate zones, from hot and cold desert to mediterranean to semi-arid, and with a sociodemographically diverse population improving the generalizability of our findings to other diverse populations across the U.S. and similar countries; however, our findings might have limited generalizability, for example, to populations in much colder or warmer climates or with markedly different sociodemographic distribution.

This study has several strengths. First, inherent to the case-crossover study design, we were able to control for time-invariant individual-level factors, including factors that might be important confounders such as gender, race and ethnicity, and chronic conditions. Second, we had a very large sample size consisting of over 1.5 million deaths and over 6 million case and control days providing statistical power to identify small odds ratios or modifying effects. Third, we evaluated multiple area-level and an individual level SES and socioenvironmental burden metrics that estimate socioenvironmental factors differently, making our conclusion more robust.

## Conclusions

In this time-stratified case-crossover study of deaths in California from 2014–2019 we found no consistent effect measure modification by census tract level socioenvironmental and socioeconomic burden or individual level SES of the mortality associations for extreme heat and PM_2.5_. However, we did find increased number of days with extreme PM_2.5_ exposure in census tracts with high socioenvironmental and socioeconomic burden. With such limited research on the joint effect of heat and PM_2.5_ exposure, more studies are needed to investigate the effect measure modification of individual and area-level socioenvironmental and socioeconomic burdens.

## Supplementary information

Supplementary Material

Supplementary Table S1

Supplementary Table S2

Supplementary Table S3

Supplementary Table S4

## Data Availability

Data on neighborhood socioenvironmental and socioeconomic data are available from the CalEnviroscreen database (https://oehha.ca.gov/calenviroscreen/report/calenviroscreen-40) and social deprivation index database (https://www.graham-center.org/maps-data-tools/social-deprivation-index.html), respectively. The mortality data were obtained from the California Department of Public Health through a Vital Statistics Application, and researchers must complete the application process on their own to obtain it.
