# Supplementary material for: Does socioeconomic and environmental burden affect vulnerability to extreme air pollution and heat? A case-crossover study of mortality in California
Source: J Expo Sci Environ Epidemiol. 2024 May 7;35(2):294–302. doi: 10.1038/s41370-024-00676-9 (PMC11540871; doi:10.1038/s41370-024-00676-9)
Supplement: Supplementary file 1 — Supplementary Material [file 41370_2024_676_MOESM1_ESM.pdf]

**Supplementary material**

**Supplementary table S1. Indicators for the socioenvironmental burden score (CalEnviroscreen score).**
See excel file

**Supplementary table S2. Indicators for the socioeconomic burden score (Social deprivation index score).**
See excel file

**Supplementary table S3. Distribution of cases and exposure level by extreme exposure category among all-cause mortality. Mean (standard deviation), unless otherwise noted.**
See excel file

**Supplementary table S4. Stratum-specific effect estimates and interaction (ixn) p-values by quantiles of socioenvironmental- (CES) and socioeconomic (SDI) burden, educational attainment, and from sensitivity analyses.**
See excel file

**Supplementary figure S1:**

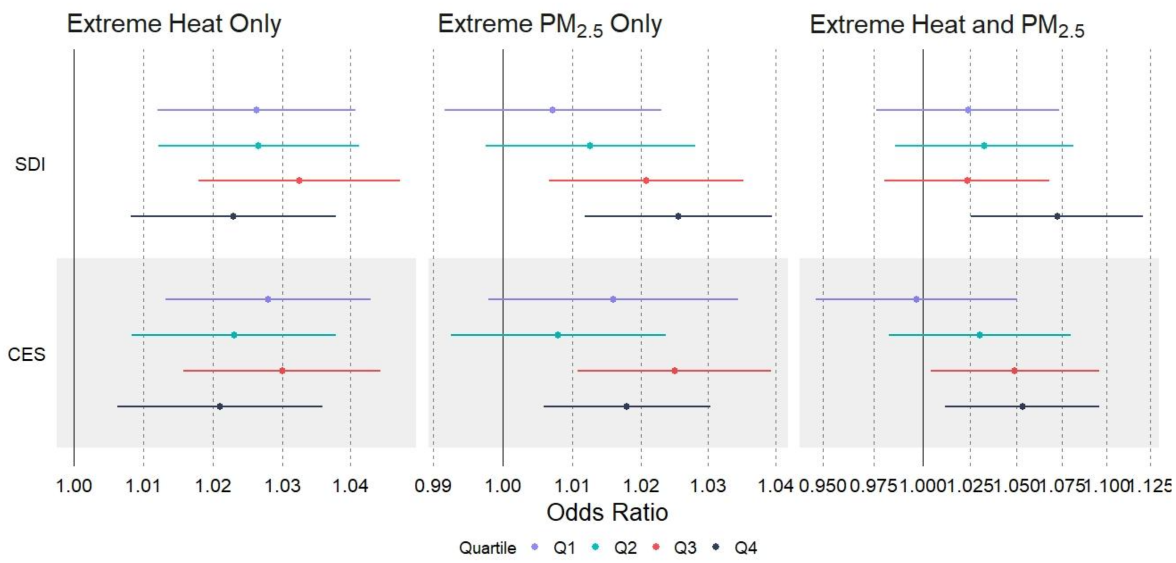


Forest plot for stratum-specific (by quartiles of socioenvironmental- and socioeconomic burden) effect estimates for the association between extreme heat, air pollution and combined heat and air pollution and respiratory mortality for lag1 extreme exposure over the 90th percentile.

**Supplementary figure S2:**

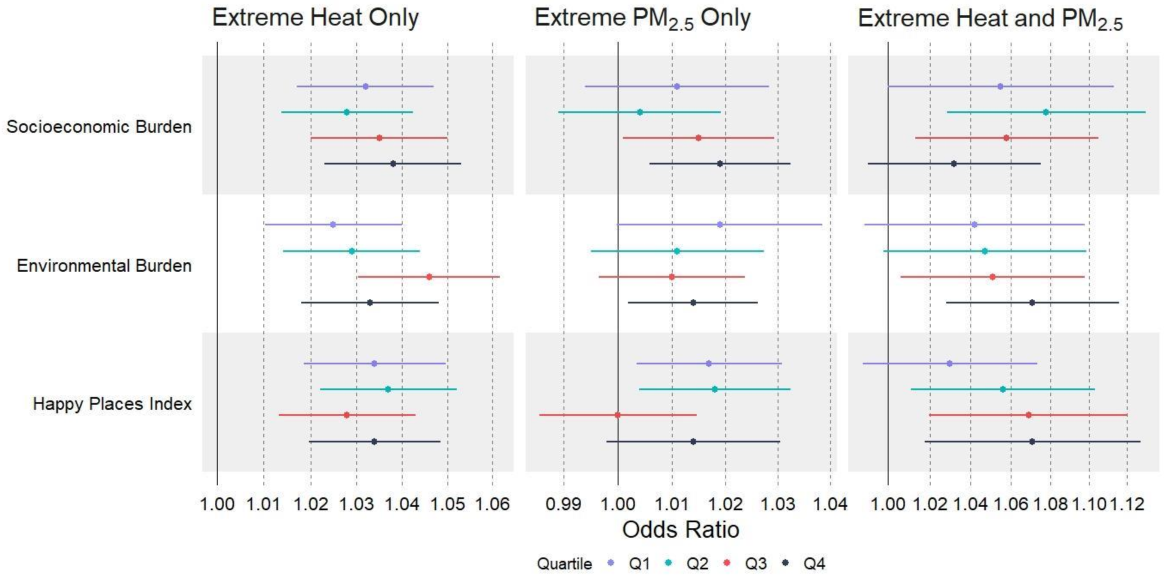


Forest plot for stratum-specific effect estimates for the association between extreme heat, air pollution and combined heat and air pollution and all-cause mortality for extreme exposure over the 90th percentile. Strata are defined based on quartiles of socioeconomic burden from the CES instead of the SDI, environmental burden alone from the CES (i.e without socioeconomic burden) and the socioenvironmental burden from the happy places index (HPI) instead of the CES.

**Supplementary file S1. Reproducibility checklist.**

SPRINGER NATURE

Corresponding Author name: Mehjar Azzouz

Manuscript Number: JESEE-23-4389.R2

**Reporting Checklist**

This checklist is used to ensure the quality, transparency, and reproducibility of published results. We require authors attest that these components have been considered and addressed.

| **Exposure Assessment Guiding Principle** | **Yes/No/Not Applicable** |
|---|---|
| Has the method to estimate exposure been described clearly? | Yes |
| Has the exposure assessment method been validated/evaluated as a proxy for exposure and is its validity or agreement with other methods described? | Yes |
| Is the time period over which the exposure assessment method is considered to be a proxy for exposure appropriate for the research question? | Yes |
| If exposure is modeled or measured, were all critical potential routes and sources of exposure considered? | Yes |
| If exposure is modeled, how does it vary over space and time and are necessary historical data incorporated? | Yes |
| If biomarkers are used as indicators of exposure, could the biomarker measurement have been affected by the outcome (i.e., reverse causality)? | NA |
| Are the strengths and weaknesses of the exposure approach detailed and discussed? | Yes |
